# Utility task vehicle crashes and injuries in Iowa

**DOI:** 10.1186/s40621-025-00595-9

**Published:** 2025-07-29

**Authors:** Parker R. Sternhagen, Christopher D. Monson, Gerene M. Denning, Charles A. Jennissen

**Affiliations:** 1 University of Iowa College of Liberal Arts and Sciences, Iowa City, IA USA; 2 Division of Critical Care, Department of Pediatrics, University of Rochester, 601 Elmwood Ave, Box 667, Rochester, NY USA; 3 Department of Emergency Medicine, Roy J. and Lucille A. Carver College of Medicine, University of Iowa, Iowa City, IA USA; 4 Stead Family Department of Pediatrics, Roy J. and Lucille A. Carver College of Medicine, University of Iowa, Iowa City, IA USA; 5 University of Iowa Hospitals and Clinics, 200 Hawkins Dr, Iowa City, IA 52242 USA

**Keywords:** Passengers, Recreational off-highway vehicle, Road, Rollover, Seatbelt, Safety, Side-by-side vehicle, Utility task vehicle, Youth

## Abstract

**Background:**

Utility task vehicles (UTVs) are increasing in popularity and have outsold all-terrain vehicles (ATVs) in recent years. However, there are few publications related to UTV crashes and injuries. Our objective was to describe UTV crash and injury epidemiology in the state of Iowa.

**Methods:**

A comprehensive database of off-highway vehicle events from the Iowa Department of Transportation, State Trauma Registry and Department of Natural Resources, as well as from newspaper articles was created and used to evaluate Iowa UTV crashes and injuries from 2002 to 2019. Frequencies and contingency table analysis was performed with IBM SPSS Statistics (Version 27).

**Results:**

UTV crashes involving 448 injured individuals of all ages were identified with increasing numbers over time. Children < 16 years were 31% and those 16–17 years were 8.3%. Among all victims, 69% were male and one-third were passengers. Only 10% and 32% were wearing helmets and seatbelts, respectively. Of those tested, 13% were positive for alcohol. One-fifth involved a collision with another motor vehicle, 11% were collisions with an object, and most (70%) were non-collision events (e.g., rollovers). In nearly two-thirds of cases, the victim was ejected. In a quarter, the person was hit/pinned by the vehicle. Of those with known location, 61% occurred on public roadways. In 6% of cases, the individual died. Children < 16 years had higher proportions than those older of being a passenger (52% vs. 24%, *p* < 0.001), having been in a non-collision event (77% vs. 66%, *p* = 0.035), and of being hit/pinned by the vehicle (41% vs. 19%, *p* < 0.001). Passengers had lower proportions that were wearing seatbelts (23% vs. 36%, *p* = 0.029) and higher proportions involved in non-collision events (90% vs. 59%, *p* < 0.001). Higher proportions in off-road crashes (55% vs. 9%, *p* < 0.001) and in non-collision events (33% vs. 8%, *p* < 0.001) were hit/pinned by the vehicle. Roadway crashes and ejected victims both had greater percentages with abnormal Glasgow Coma Scale (head injury) scores and intensive care unit admission.

**Conclusions:**

UTV crashes and injuries are increasing in frequency and often associated with severe injuries. Driving on public roads and being ejected were both associated with more severe outcomes/injuries.

## Background

Side-by-side vehicles are off-highway vehicles that have become increasingly popular over the past 25 years with escalating crash-related deaths and injuries [1–3]. Unlike all-terrain vehicles (ATVs) which have a straddle seat and handlebars, side-by-side vehicles have bucket/bench seating, a steering wheel, and foot controls for breaking and acceleration. Although they were previously called recreational off-highway vehicles (ROVs), most consumers use the term utility task vehicle (UTV) for side-by-sides and many manufacturers are now following this convention in their marketing. Accordingly, we will also use the term “UTV” when referring to “ROVs” in this manuscript.

Most UTVs travel highway speeds, some up to 85 mph [4], which has helped fuel the vehicle’s popularity. UTV sales are now more than double those of ATVs with an estimated 565,00 UTVs versus 240,000 ATVs sold in North America in 2024 [5]. All UTVs that travel at least 30 mph are required to have a roll-over protective structure (ROPS) and either seatbelts or harnesses at each seating position to keep riders within the zone of protection afforded by the ROPS.

Almost all UTVs are designed for operators 16 years of age and older. This is reinforced by manufacturers through vehicle stickers and in owner’s manuals. Moreover, manufacturers recommend one should be at least 12 years of age or be able to sit with their back against the seat with their feet flat on the vehicle floor before riding as a passenger in UTVs. Unfortunately, many parents allow their children to operate and ride in these vehicles despite manufacturer warnings. A study of over 4,000 Iowa students 9–18 years old found that more than two-thirds had ridden and/or driven a UTV in the past year with approximately two-fifths riding in a vehicle at least weekly [6].

Unlike ATVs, there have been few studies that have examined UTV crash and injury epidemiology. The U.S. Consumer Product Safety Commission (CPSC) has been tracking ATV-related deaths and estimated injuries for decades but just recently started including data related to UTVs [2]. In their most recent report published in May 2023, the Commission identified 643 deaths due to UTVs from 2017 to 2019, accounting for nearly a third of off-highway vehicle deaths in their database [2]. Other studies have examined UTV data of patients treated at the University of Iowa, collected by the Fatality Analysis Reporting System, and of newspaper clippings from nine Midwest and Great Plains States [1, 3, 7]. Several studies have reviewed specific UTV-related injuries including upper extremity injuries evaluated at the University of Utah [8] and orthopedic injuries treated at Loma Linda University Health [9].

Similar to ATVs after their introduction, the public health concerns related to UTVs have been growing as associated deaths and injuries increase. The trauma experienced by children and adolescents from UTVs has been particularly troubling. Identifying risk factors associated with UTV crashes and the severe injuries they produce is crucial for developing injury prevention strategies. Our objective was to examine the epidemiology of UTV crashes and injuries in Iowa and to identify associated risk factors.

## Methods

A retrospective study utilizing our Iowa off-road vehicle (ORV) surveillance database was performed on UTV-related crashes and injuries that occurred from January 1, 2002, to December 31, 2019. The study was approved by the University of Iowa Institutional Review Board.

### ORV surveillance database

The Iowa ORV surveillance database was previously created by comprehensively collecting ORV crashes and injuries from the Iowa Department of Transportation (DOT), the Iowa State Trauma Registry (STR) and the Iowa Department of Natural Resources (DNR), as well as from newspaper articles from 2002 to 2019 [10–14]. Data were obtained in compliance with all local, state and federal regulations. Newz Group, a news monitoring service specializing in rural, local and regional coverage, was utilized to obtain press clippings of ORV crashes [7].

A standardized coding system was developed as previously described [10–14]. This included multiple strategies to identify UTV crashes and injuries, methods for standardized coding of each database’s narratives, and linking of an individual’s data across databases using Link Plus Version 2.0. Finally, a merged dataset was created by identifying matching records in the following sequence: all DOT cases, STR cases without DOT matches, DNR cases without DOT and STR matches, and newspaper clippings without DOT, STR, and DNR matches. All cases identified were included, even if found in just one data source. Only individuals reported as operators or passengers of the vehicle were included in the study; pedestrians were excluded.

## Study variables

Demographic variables included sex (male, female) and age (years). For bivariate analysis, age was grouped into two categories; <16 years (not old enough to operate UTVs per manufacturer’s recommendations) and ≥ 16 years. Crash occurrence variables were Weekday (Monday-Thursday) vs. Weekend (Friday-Sunday), and Season including Winter (December-February), Spring (March-May), Summer (June-August), and Fall (September-November). Other variables included Seating Position (operator, passenger), Location (off-road vs. public roadway), Seatbelt Use (yes, no), Helmet Use (yes, no), and Alcohol Status (tested positive, tested negative). The Light Condition variable reflected light conditions at the time of the crash with Day representing daylight hours and Night including the time from dusk (starting 30 min before sunset) to dawn (30 min after sunrise).

The Crash Mechanism variable included three categories: Motor Vehicle Collision (where the UTV was in a collision with another motor vehicle such as a car, ATV or another UTV), UTV-Object Collision (described as the UTV colliding with an object other than a motor vehicle) and a Non-Collision Event (rollover or primary operator/passenger ejection). Other injury mechanism variables included Ejected from the Vehicle (yes, no) which could occur anytime during a crash event and Hit/Pinned by the Vehicle (yes, no) which entailed the individual being struck by the vehicle and/or pinned underneath.

Injury variables included Death (yes, no), Glasgow Coma Scale (GCS) score, Injury Severity Score (ISS), Days in ICU (0, ≥ 1 day), and Days in Hospital (1, ≥ 2 day). GCS provides an evaluation of a patient’s level of consciousness as an indicator of brain injury. The GCS was dichotomized for analysis as GCS = 15 (normal) or GCS < 15 (abnormal). ISS is a commonly used injury severity scoring system for trauma patients for which a score of > 15 indicates a severe injury. For analysis, ISS was dichotomized as ≤ 15 or > 15.

### Data analysis

Descriptive analyses (frequencies) and contingency table analyses were performed using SPSS (Version 27). Some additional bivariate analysis including chi square and Fisher’s exact test were conducted using the Vassar statistical calculations website (http://vassarstats.net/). Fisher’s Exact test was performed when expected cell size values were < 5. Simple linear regression was performed with Graphpad prism to evaluate the change in injuries and crashes over time. Results were defined as statistically significant with a two-tailed P-value < 0.05. Missing data were not included in frequency or bivariate analysis.

## Results

**Fig. 1:**
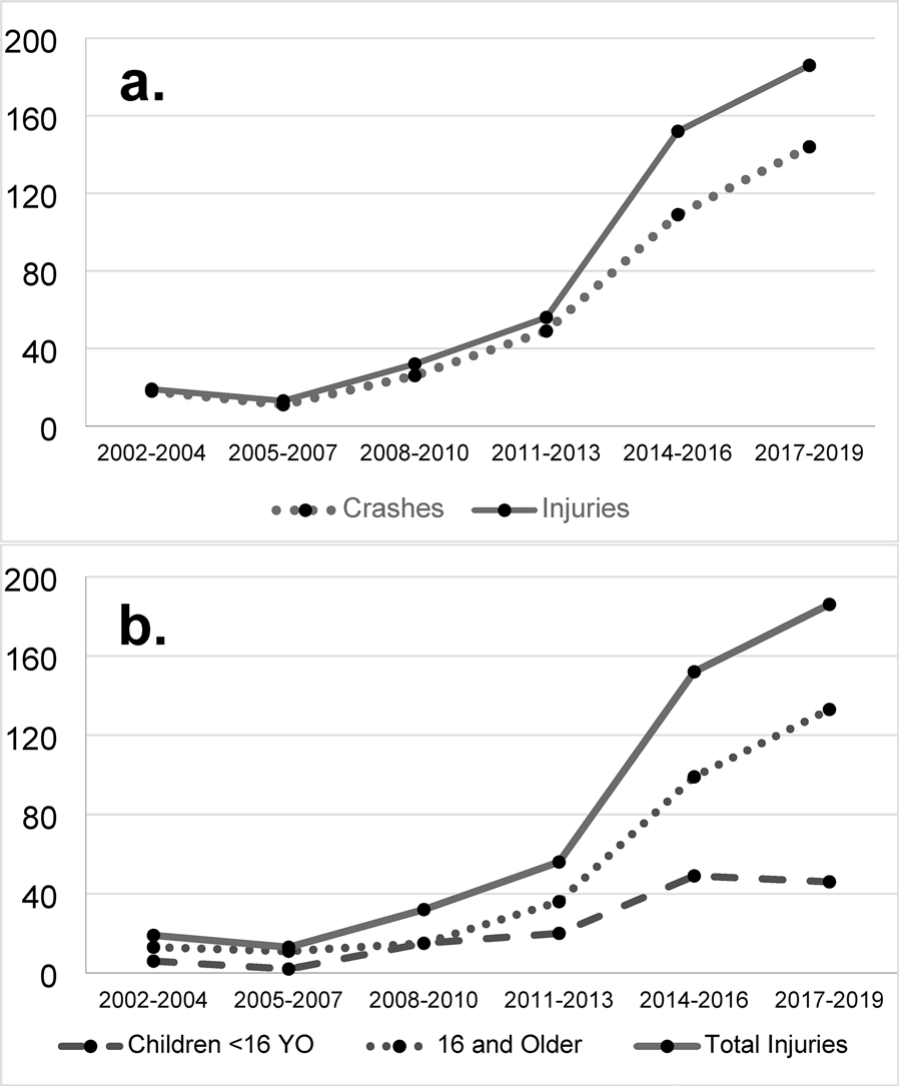
Graph of (**a**) Iowa UTV-related injuries (solid line) and crashes (dotted line) and (**b**) Iowa UTV-related injuries including total (solid line), those 16 years and older (dotted line) and those < 16 years old (dashed line) from 2002–2019. Values are grouped by three-year intervals. Data are from our Iowa off-road vehicle (ORV) surveillance database. The database combines data from the Iowa Department of Transportation (DOT), the Iowa State Trauma Registry (STR), the Iowa Department of Natural Resources (DNR) and newspaper clippings of Iowa off-road vehicle crashes over the study period

From 2002 to 2019, 458 injured individuals in 357 UTV crashes were identified in the Iowa ORV Database. Increasing numbers of injuries and crashes occurred during the time period. See Fig. 1. Simple linear regression shows the slopes for injuries to be 36.5 (95% CI 15.0-57.9) and for crashes 27.1 (95% CI 11.9–42.3) indicating the slopes are significantly different from zero and increasing over time (*p* = 0.009 and *p* = 0.008, respectively).

The majority of those injured were male (69%). See Table 1. Nearly two-fifths (39%) were ≤ 17 years, and almost one-third (31%) were 15 years of age and younger. Just over half were injured from Friday-Sunday (51%), and 44% occurred during the summer months (June-August). Of those injured, about two-thirds were operators (67%) while a third (33%) were passengers in the UTV. Over three-fifths (61%) were injured during an event on a public roadway. For those with documentation, over two-thirds of those injured (68%) were not wearing a seatbelt or harness at the time of their crash; 90% were not wearing a helmet. Of those 16 years and older tested (225/309, 73%), 13% (40/225) were positive for alcohol. More than a quarter of injuries (27%) occurred at night. Most (70%) were injured in a non-collision event (e.g., rollover), and the rest were involved in collisions with another motor vehicle (19%) or other objects (11%). For victims where it could be determined, nearly two-thirds (65%) were ejected from the vehicle and over a quarter (26%) were hit or pinned by the UTV. Twenty-eight individuals, 6% of victims, died of their injuries.

## Males vs. females

A higher proportion of injured females were UTV passengers as compared to males (50% vs. 24%), *p* < 0.001. See Table 2. Females also had greater percentages injured in non-collision events (78% vs. 66%) and lower proportions injured in collisions with other objects (5% vs. 14%), *p* = 0.011.

**Table 1:** Variable frequencies for individuals injured in utility task vehicle (UTV) crashes/events in Iowa from 2002-2019 (N = 458)

	n (Col %)^a^		n (Col %)^a^
Variable (Missing)		Variable (Missing)	
Sex (10)		Seatbelt use (161)	
Male	309 (69)	Yes	95 (32)
Female	139 (31)	No	202 (68)
Age (13)		Helmet use (167)	
<6 YO	14 (3)	Yes	29 (10)
6–11 YO	37 (8)	No	262 (90)
12–15 YO	87 (20)	Alcohol status (157)	
16–17 YO	37 (8)	Positive (Yes)	40 (13)
18–30 YO	111 (25)	Negative (No)	261 (87)
31–45 YO	76 (17)	Light Condition (151)	
45–60 YO	45 (10)	Day	224 (73)
>60 YO	38 (9)	Night	83 (27)
Weekday/weekend (0)		Crash mechanism (36)	
Weekday	224 (49)	Motor vehicle collision	82 (19)
Weekend	234 (51)	UTV-object collision	45 (11)
Season (0)		Non-collision event	295 (70)
Winter	49 (11)	Ejected from vehicle (181)	
Spring	110 (24)	Yes	181 (65)
Summer	202 (44)	No	96 (35)
Fall	97 (21)	Hit/pinned by vehicle (168)	
Seating position (70)		Yes	74 (26)
Operator	259 (67)	No	216 (74)
Passenger	129 (33)	Victim killed (5)	
Location (85)		Yes	28 (6)
Off-road	146 (39)	No	425 (94)
Public roadway	227 (61)		

^a^The sum of n may not equal the total Group N due to missing values

### Children < 16 years vs. 16 years and older

Relative to victims 16 years of age and older, children < 16 years were a higher percentage of those injured on UTVs during the summer months (58% vs. 38%) and of those who were passengers (52% vs. 24%), both *p* < 0.001. See Table 2. However, nearly half (48%) of children < 16 years were operators of the UTV at the time of the crash. Youth < 16 years old also had lower percentages who were injured on a public roadway as compared to those ≥ 16 years (52% vs. 65%), *p* = 0.019; still, over half of children < 16 years were on public roads in a UTV at the time of their injury.

Additionally, as compared to older victims, children < 16 years of age had lower proportions of crashes occurring at night (10% vs. 33%), *p* < 0.001, and under the influence of alcohol (0% vs. 18%), *p* < 0.001. With respect to mechanism, crashes involving youth < 16 years had a higher percentage that were non-collision events (77% vs. 66%) and a lower percentage that were collisions with another motor vehicle (13% vs. 23%), *p* = 0.035. Moreover, children < 16 years had higher proportions that had been hit/pinned by the vehicle relative to victims ≥ 16 years of age (41% vs. 19%), *p* < 0.001.

## Passengers vs. operators

In addition to differences seen in the proportion of passengers and operators by sex and by age, higher proportions of passengers than operators were injured in the summer (53% vs. 38%, *p* = 0.009) and were not wearing seatbelts (77% vs. 64%, *p* = 0.029). See Table 3. Passengers also had higher percentages injured in non-collision events as compared to operators (90% vs. 59%), *p* < 0.001.

**Table 2:** Bivariate analyses for age and sex of individuals injured in utility task vehicle (UTV) crashes/events in Iowa from 2002-2019 (N = 458)

	Age			Sex		
	< 16 Years n (Col %)^a^	≥16 Years n (Col %)^a^	p value	Male n (Col %)^a^	Female n (Col %)^a^	p value
Group N	139	309		309	139	
Sex						
Male	93 (68)	214 (70)	0.700	--	--	.
Female	44 (32)	93 (30)		--	--	
Weekday/weekend						
Weekday	70 (50)	147 (48)	0.585	151 (49)	68 (49)	0.992
Weekend	69 (50)	162 (52)		158 (51)	71 (51)	
Season						
Winter	7 (5)	41 (13)	< 0.001	38 (12)	8 (6)	0.050
Spring	29 (21)	78 (25)		80 (26)	28 (20)	
Summer	80 (58)	116 (38)		126 (41)	71 (51)	
Fall	23 (17)	74 (24		65 (21)	32 (23)	
Seating position						
Operator	51 (48)	207 (76)	< 0.001	201 (76)	58 (50)	< 0.001
Passenger	55 (52)	65 (24)		65 (24)	57 (50)	
Location						
Off-road	52 (48)	89 (35)	0.019	103 (41)	37 (33)	0.173
Public roadway	57 (52)	168 (65)		151 (59)	75 (67)	
Seatbelt use						
Yes	23 (28)	72 (34)	0.307	64 (30)	31 (38)	0.170
No	59 (72)	138 (66)		150 (70)	50 (62)	
Helmet use						
Yes	5 (6)	24 (12)	0.187	21 (10)	8 (10)	0.975
No	74 (94)	183 (88)		189 (90)	71 (90)	
Alcohol status						
Positive (Yes)	0 (0)	40 (18)	< 0.001^b^	31 (16)	9 (9)	0.093
Negative (No)	73 (100)	185 (82)		167 (84)	94 (91)	
Light condition						
Day	71 (90)	146 (67)	< 0.001	138 (70)	79 (79)	0.100
Night	8 (10)	72 (33)		59 (30)	21 (21)	
Crash mechanism						
Motor vehicle collision	16 (13)	66 (23)	0.035	60 (21)	22 (17)	0.011
UTV-object collision	13 (10)	32 (11)		39 (14)	6 (5)	
Non-collision event	99 (77)	189 (66)		189 (66)	100 (78)	
Ejected from vehicle						
Yes	53 (65)	127 (66)	0.920	128 (66)	53 (64)	0.693
No	28 (35)	65 (34)		65 (34)	30 (36)	
Hit/pinned by vehicle						
Yes	32 (41)	40 (19)	< 0.001	52 (27)	21 (22)	0.388
No	46 (59)	168 (81)		142 (73)	74 (78)	

^a^The sum of n for a variable may not equal the total Group N due to missing values

^b^p-value was calculated from Fisher’s Exact Test

## Off-road vs. public roadways

As stated earlier, lower proportions of injured youth < 16 years were on roadways as compared to those older. See Table 3. In addition, the proportion of all victims wearing a helmet was lower on roadways than off-road (1% vs. 24%), *p* < 0.001. Individuals injured off-road had greater proportions injured in non-collision events (87% vs. 52%) and lower proportions in a crash with another motor vehicle (0% vs. 36%), *p* < 0.001. A higher percentage werealso hit/pinned by the UTV off-road than on public roadways (55% vs. 9%), *p* < 0.001.

### Crash mechanisms

In addition to differences seen in the proportions of crash mechanisms by sex, age, seating position and location, those injured in a collision with another motor vehicle had the highest percentage reportedly wearing a seatbelt, but that was still less than half (48%), *p* = 0.006. See Table 4. Helmet use was higher in those injured in a non-collision event than for other crash mechanisms, *p* < 0.001, but that was still only 14%. None of those in a collision with a motor vehicle were positive for alcohol whereas the percentage was higher for those in a non-collision event (16%) and highest in those who collided with an object (26%), *p* < 0.001. Most collisions with a motor vehicle occurred during the day (94%) which was higher than for non-collision events (68%) and for collisions with an object (58%), *p* < 0.001. Non-collision events had the highest proportion of injured individuals struck/pinned by the UTV (33%) which was higher than for collisions with an object (21%) and with another motor vehicle (1%), *p* < 0.001.

**Table 3:** Bivariate analyses of seating position and off-road/public roadway for individuals injured in utility task vehicle (UTV) crashes/events in Iowa from 2002-2019 (N = 458)

	Seating position			Off-road/public roadway		
	Operator n (Col %)^a^	Passenger n (Col %)^a^	p value	Off-road n (Col %)^a^	Public roadway n (Col %)^a^	p value
Group N	259	129		146	227	
Sex						
Male	201 (78)	65 (53)	< 0.001	103 (74)	151 (67)	0.173
Female	58 (22)	57 (47)		37 (26)	75 (33)	
Age						
<16 yrs	51 (20)	55 (46)	< 0.001	52 (37)	57 (25)	0.019
16 and older	207 (80)	65 (54)		89 (63)	168 (75)	
Weekday/weekend						
Weekday	122 (47)	71 (55	0.141	70 (48)	112 (49)	0.795
Weekend	137 (53)	58 (45)		76 (52)	115 (51)	
Season						
Winter	34 (13)	6 (5)	0.009	17 (12)	22 (10)	0.853
Spring	67 (26)	28 (22)		38 (26)	54 (24)	
Summer	99 (38)	69 (53)		62 (42)	101 (44)	
Fall	59 (23)	26 (20)		29 (20)	50 (22)	
Location						
Off-road	78 (36)	34 (34)	0.758	--	--	--
Public roadway	140 (64)	66 (66)		--	--	
Seatbelt use						
Yes	67 (36)	20 (23)	0.029	22 (27)	48 (29)	0.795
No	118 (64)	67 (77)		59 (73)	119 (71)	
Helmet use						
Yes	18 (10)	6 (7)	0.420	19 (24)	2 (1)	< 0.001^b^
No	166 (90)	82 (93)		59 (76)	167 (99)	
Alcohol status						
Positive	24 (13)	12 (14)	0.823	8 (14)	21 (11)	0.591
Negative	159 (87)	73 (86)		51 (86)	170 (89)	
Light condition						
Day	138 (72)	71 (74)	0.759	47 (65)	142 (76)	0.097
Night	53 (28)	25 (26)		25 (35)	46 (24)	
Crash mechanism						
Motor vehicle collision	69 (28)	5 (4)	< 0.001	0 (0)	79 (36)	< 0.001
UTV-object collision	31 (13)	7 (6)		17 (13)	24 (11)	
Non-collision event	144 (59)	110 (90)		117 (87)	115 (52)	
Ejected from vehicle						
Yes	119 (68)	45 (62)	0.335	42 (58)	111 (68)	0.132
No	56 (32)	28 (38)		31 (42)	53 (32)	
Hit/pinned by vehicle						
Yes	34 (19)	22 (25)	0.298	37 (55)	17 (9)	< 0.001
No	141 (81)	66 (75)		30 (45)	175 (91)	

^a^The sum of n for a variable may not equal the total Group N due to missing values

^b^p-value was calculated from Fisher’s Exact Test

### Light conditions

As mentioned earlier, injured children < 16 years had lower proportions riding at night than those older. Other significant findings included those injured at night had lower seatbelt use (13/62, 21% vs. 62/155, 40%, *p* = 0.008) and higher proportions positive for alcohol (18/60, 30% vs. 13/187, 7%, *p* < 0.001).

### Injury severity indicators

Injury data were available for 220 hospitalized individuals through the Iowa STR. An abnormal Glasgow Coma Scale (GCS) score (< 15) indicating an altered mental status was identified in 11% of hospitalized victims and a moderate or higher Injury Severity Scale score (> 15) was noted in 12%. See Table 5. Almost two-fifths (37%) required at least one day in the intensive care unit (ICU) and ICU stays ranged from 1 to 30 days. Two-thirds (66%) were hospitalized for more than one day and length of stay ranged from 1 to 80 days.

**Table 4:** Bivariate analyses of crash mechanisms for individuals injured in utility task vehicle (UTV) crashes/events in Iowa from 2002-2019 (N = 458)

	Motor vehicle collision *n* (Col %)^a^	UTV-object collision *n* (Col %)^a^	Non-collision event *n* (Col %)^a^	*p* value
Group N	82	45	295	
Sex				
Male	60 (73)	39 (87)	189 (65)	0.011
Female	22 (27)	6 (17)	100 (35)	
Age				
<16 yrs old	16 (20)	13 (29)	99 (34)	0.035
16 yrs and older	66 (80)	32 (71)	189 (66)	
Weekday/weekend				
Weekday	46 (56)	26 (58)	136 (46)	0.134
Weekend	36 (44)	19 (42)	159 (54)	
Season				
Winter	12 (15)	4 (9)	31 (11)	0.647
Spring	17 (21)	10 (22)	79 (27)	
Summer	33 (40)	20 (44)	131 (44)	
Fall	20 (24)	11 (24)	54 (18)	
Seating position				
Operator	69 (93)	31 (82)	144 (57)	<0.001
Passenger	5 (7)	7 (18)	110 (43)	
Location				
Off-road	0 (0)	17 (41)	117 (50)	<0.001
Public roadway	79 (100)	24 (59)	115 (50)	
Seatbelt use				
Yes	31 (48)	9 (29)	50 (27)	0.006
No	33 (52)	22 (71)	136 (73)	
Helmet use				
Yes	1 (2)	1 (3)	25 (14)	0.005^b^
No	63 (98)	28 (97)	158 (86)	
Alcohol status				
Positive	0 (0)	9 (26)	29 (16)	<0.001^b^
Negative	72 (100)	26 (74)	148 (84)	
Light condition				
Day	68 (94)	21 (58)	123 (68)	< 0.001
Night	4 (6)	15 (14)	57 (32)	
Ejected from vehicle				
Yes	42 (63)	22 (69)	113 (65)	0.839
No	25 (37)	10 (31)	61 (35)	
Hit/pinned by vehicle				
Yes	1 (1)	7 (21)	66 (33)	< 0.001
No	69 (99)	26 (79)	114 (67)	

^a^The sum of n for a variable may not equal the total Group N due to missing values

^b^p-value was calculated from Fisher’s Exact Test

Those who were ejected from the UTV had higher proportions with an abnormal GCS (19% vs. 0%), *p* = 0.045, and had a higher proportion spending at least one day in the ICU (58% vs. 8%), *p* = 0.027. See Table 5. In addition, patients whose injury occurred on a public roadway had higher percentages with an abnormal GCS (25% vs. 6%), *p* = 0.008, and had greater percentages that had at least one day in the ICU (82% vs. 27%), *p* = 0.003.

**Table 5:** Bivariate analyses of injury severity indicators in relationship to being ejected from the vehicle and the location of the crash event for individuals injured in utility task vehicle (UTV) crashes/events in Iowa from 2002-2019 (N = 220 from Iowa Trauma Registry)

	Total n (Col %)^a^	Ejected from vehicle			Location		
		Yes n (Col %)^a^	No n (Col %)^a^	p value	Off-road n (Col %)^a^	Public Roadway n (Col %)^a^	p value
Group N		58	34		101	41	
GCS							
GCS = 15	144 (89)	34 (81)	25 (100)	0.045^b^	66 (94)	24 (75)	0.008^b^
GCS < 15	18 (11)	8 (19)	0 (0)		4 (6)	8 (25)	
ISS							
ISS ≤ 15	186 (88)	48 (84)	30 (94)	0.315^b^	85 (89)	31 (79)	0.124
ISS > 15	26 (12)	9 (16)	2 (6)		10 (11)	8 (21)	
Days in ICU							
0 days	42 (63)	5 (42)	11 (92)	0.027^b^	22 (73)	2 (18)	0.003^b^
1 or more days	25 (37)	7 (58)	1 (8)		8 (27)	9 (82)	
Days in hospital							
1 day	31 (34)	4 (21)	3 (25)	1.000^b^	11 (28)	10 (48)	0.133
2 or more days	59 (66)	15 (79)	9 (75)		28 (72)	11 (52)	

GCS: Glasgow Coma Scale, ISS: Injury Severity Score

^a^The sum of n for a variable may not equal the total Group N due to missing values

^b^p-value was calculated from Fisher’s Exact Test

## Discussion

Our study examined the epidemiology of UTV crashes and injuries in Iowa utilizing our unique statewide ORV surveillance database. There was a striking increase in crashes and injuries over the study period, similar to that observed by other investigators [1–3]. Seatbelt and helmet use, critical for preventing serious injury in UTV crashes, were very limited. Most individuals were injured in non-collision events, two-thirds were ejected and one-quarter were struck/pinned by the vehicle. Over three-fifths were injured on public roadways. Passengers made up a significant proportion of those injured. UTV-related injuries were often severe and associated with prolonged ICU and hospital stays, as well as fatalities. A particular concern was the high proportion of those injured being children.

### Youth

Overall, about two-fifths of UTV-injured individuals were youth < 18 years. Moreover, almost one-third were < 16 years of age and, of these, nearly half were the UTV operator at the time of the injury. UTV manufacturers warn that these vehicles should not be operated by anyone under 16 years and that passengers be at least 12 years of age. However, it is not clear to what extent the high levels of unsafe behavior result from parents being unaware of these warnings, or aware but not heeding them.

Other studies have also noted significant proportions of injured crash victims being children < 16 years [1, 7, 15]. Children are reportedly only 14-18% of all UTV drivers yet make up a third to half of UTV injuries [1, 3, 7, 16]. Of the more than two-thirds of Iowa students that had ridden in a UTV in the past year, 29% reported having at least one crash during that time [6]. A newspaper report study of nine Midwest and Great Plains states showed UTV crashes were about 2.5 times more likely to involve a child than ATV crashes [7].

### Rollovers

Children < 16 years had higher proportions than adults injured in non-collision events, mostly rollovers. This may partially explain why children had greater percentages than adults who were hit/pinned by the vehicle, even though their seatbelt use was not significantly different. UTVs typically weigh 1000–2000 pounds and are challenging to lift off pinned individuals. Victims can die of traumatic injuries to the head or vital organs, by compressive asphyxiation, and/or can suffer extremity amputations [3, 8, 9, 17].

Other studies have similarly found that rollovers account for the majority of UTV crashes [1, 3, 15]. CPSC data from 2003 to 2011 showed that rollovers frequently occur while turning and at speeds < 20 mph [15, 18]. Although a rollover can be initiated through a collision or when traveling on slopes, of the two-thirds of CPSC fatalities (*N* = 224) involving rollovers, 38% occurred on flat terrain [15]. Similar to ATVs, UTVs have a relatively high center of gravity and narrow track making them highly susceptible to rollovers [15, 19, 20].

### Proper restraint

Two-thirds of those injured in UTV crashes were ejected from the vehicle. Ejected victims had significantly higher percentages with an abnormal GCS and ICU admission. A CPSC database study of UTV fatalities found that 86% of victims were partially or fully ejected with three-quarters being struck by the vehicle and high proportions being pinned [15]. Other studies have similarly shown high proportions of patients being ejected and struck/pinned by the UTV [1, 3, 7].

Our study found that just 32% of those injured were wearing a seatbelt at the time of the crash. Other studies have found even lower proportions, ranging from 7 to 28% [1, 3, 7, 8, 15, 17]. A study of Iowa adolescent students found 37% reporting they never or almost never wore a seatbelt while in UTVs [6]. Proper restraint for every person during every ride is a critical UTV safety behavior. However, UTV seatbelts are not designed for children under 12 years and are not tested for use with child safety seats and booster seats limiting their protective function for youth.

### Public roadways

UTVs are not designed for public roadways. Their off-road design includes low pressure tires with knobby treads meant to grab off-road terrain and they can have very unpredictable interactions with public roadway surfaces. Manufacturers strongly recommend against riding UTVs on public roadways [21] and warn consumers in the owner’s manuals and with stickers on the vehicles.

However, where location could be determined, over 60% of study crashes occurred on public roadways. For children < 16 years of age, one quarter were injured on public roads. Other studies have found significant proportions of UTV-related injuries occurring on public roadways, ranging from 29 to 56% [1, 3, 7]. In addition, the majority of UTV roadway injuries in our study did not involve a collision with another motor vehicle. Other studies have shown that about four-fifths of UTV-related public roadway crashes were single vehicle crashes not involving another motor vehicle [1, 7]. Moreover, victims injured on public roads in our study had higher percentages with abnormal GCS scores and hospitalizations requiring ICU care.

The public needs to be better educated about the hazards of UTV roadway riding. Unfortunately, there has been a disturbing trend across the U.S. of cities, counties and states passing legislation allowing the recreational and transportation-related use of ORVs (including ATVs and UTVs) on public roads [14, 22, 23]. An Iowa study showed that counties passing ordinances allowing ORVs on public roads had a nearly 60% higher crash rate as compared to before it was passed and as compared to counties that had never passed a law [14]. Instead of passing legislation against manufacturer’s recommendations that make the public less safe, all those interested in preventing ORV-related deaths and injuries should be advocating for the passage and enforcement of laws that prohibit ORVs on public roads.

### Limitations

Limitations of our study include incomplete capture of crash and injury records as well as missing and/or incomplete documentation of study variables in our database which may have impacted our results. Another limitation is that moderate and severe crashes and injuries have a greater probability of being included in the data sources used in the study as compared to less severe crashes and injuries that did not require medical attention or were treated in the outpatient setting. Deaths that occurred but were not treated at a medical facility may also be missing. We identified some potential sampling bias in alcohol testing with higher proportions of UTV operators on public roads being tested, so the involvement of alcohol may be underestimated in the study due to these and other limitations. Moreover, lack of vehicle information and confusion on the terminology of ORVs might have led some UTVs being misidentified as ATVs and excluded from the study. Narratives were carefully examined to correctly categorize ORVs in the database in order to minimize this limitation. In addition, our study was limited to UTV victims injured in the state of Iowa and does not necessarily reflect the experience of other states. Despite these limitations and the uncertain generalizability of our results, many of our findings are similar to that of the few UTV reports published and builds on the highly limited information currently available.

## Conclusions

UTV crashes and injuries are increasing in frequency and are often associated with severe injuries. Injured victims in the study were often violating basic UTV safety rules. Children < 16 years and < 12 years who are not recommended by manufacturers to be UTV operators and passengers, respectively, were nearly one-third of those injured. Ejected UTV occupants and those driving on public roads had more severe outcomes. Decreasing UTV-related deaths and injuries will require widespread efforts to educate the public on UTV safety, and the passage and enforcement of evidence-based safety legislation including seatbelt use requirements, youth age restrictions, and the prohibition of UTVs (except for occupational purposes) on public roads.

## Data Availability

Data and materials are available to other parties for research purposes after a data sharing agreement plan is agreed to and signed. Those interested should contact the corresponding author.

## Abbreviations

ATV: All-terrain vehicle
CPSC: Consumer Product Safety Commission
DNR: Department of Natural Resources
DOT: Department of Transportation
ED: Emergency department
e.g.: Exempli gratia (for example)
GCS: Glasgow Coma Scale
ISS: Injury severity score
ICU: Intensive care unit
ORV: Off-road vehicle
ROV: Recreational off-highway vehicle
ROPS: Roll-over protective structure
STR: State Trauma Registry
SPSS: Statistical Package for the Social Sciences
UTV: Utility task vehicle
